# Recent developments in X-ray diffraction/scattering computed tomography for materials science

**DOI:** 10.1098/rsta.2022.0350

**Published:** 2023-10-30

**Authors:** Naomi E. Omori, Antonia D. Bobitan, Antonis Vamvakeros, Andrew M. Beale, Simon D. M. Jacques

**Affiliations:** ^1^ Finden Limited, Merchant House, 5 East St Helens Street,Abingdon OX14 5EG, UK; ^2^ Department of Chemistry, University College London, 20 Gordon Street, London WC1H 0AJ, UK; ^3^ Research Complex at Harwell, Rutherford Appleton Laboratory, Harwell Science and Innovation Campus, Didcot, Oxon OX11 0FA, UK; ^4^ Dyson School of Design Engineering, Imperial College London, London SW7 2DB, UK

**Keywords:** XRD-CT, tomography, X-ray, scattering, diffraction, chemical imaging

## Abstract

X-ray diffraction/scattering computed tomography (XDS-CT) methods are a non-destructive class of chemical imaging techniques that have the capacity to provide reconstructions of sample cross-sections with spatially resolved chemical information. While X-ray diffraction CT (XRD-CT) is the most well-established method, recent advances in instrumentation and data reconstruction have seen greater use of related techniques like small angle X-ray scattering CT and pair distribution function CT. Additionally, the adoption of machine learning techniques for tomographic reconstruction and data analysis are fundamentally disrupting how XDS-CT data is processed. The following narrative review highlights recent developments and applications of XDS-CT with a focus on studies in the last five years.

This article is part of the theme issue 'Exploring the length scales, timescales and chemistry of challenging materials (Part 2)'.

## 1. Introduction

Interrogation of material systems have benefitted greatly in the last few decades from the development of advanced chemical imaging techniques. Among these, and the topic of the present review, are X-ray diffraction scattering (XDS) computed tomography (CT) techniques. CT is a powerful imaging technique that can achieve non-destructive cross-sectional visualization of three-dimensional volumes using reconstructive mathematical algorithms on projection data. A key feature of CT is its ability to spatially resolve almost any detectable interaction from a penetrating beam in a geometrically accurate manner. Notably, this includes phenomena that can provide quantitative information pertaining to the sample's structure and chemical composition, such as coherent scatter or X-ray fluorescence (XRF).

X-ray scattering techniques are a natural complement to conventional X-ray absorption-contrast CT, being non-destructive techniques offering nanometric information on structural parameters, elemental composition and phase composition. While X-ray diffraction CT (XRD-CT) predominates as the mainstay XDS-CT technique, it has also developed to include methods such as small angle X-ray scattering CT (SAXS-CT), pair distribution function CT (PDF-CT), and hybrid techniques, the latter of which has also seen a rise in studies demonstrating the concomitant use of spectroscopy-based methods such as XRF-CT, offering even greater chemical insight. It should be noted at this point that the abbreviation ‘XDS-CT’ is used as a catchall to describe the range of related modalities currently being developed. Used here for brevity, it is not a standardized term within the field and is not a substitute term for XRD-CT, SAXS-CT, PDF-CT or any of the other modalities identified in this review.

The following review aims to highlight recent developments in XDS-CT methods with a focus on studies in the last 5 years. The first section provides a didactic overview of XDS-CT methods including a brief historical overview of X-ray CT and current capabilities of both synchrotron and laboratory-based set-ups. The second section highlights recent studies of different material systems that have benefitted from XDS-CT techniques focusing on both inorganic materials and biomaterials. The final section then explores emerging trends in the application of artificial intelligence (AI) and machine learning (ML) to XDS-CT for tomographic reconstruction, data analysis, and enhancement of both scattering patterns and reconstructedimages.

### (a) Technique overview

#### (i) A brief history

Necessarily predating advanced XDS-CT techniques was the development of absorption contrast X-ray radiography and its natural evolution absorption-contrast X-ray CT, conventionally referred to as ‘X-ray CT' (XR-CT) or in certain circles simply ‘CT' [1]. Image contrast in absorption contrast X-ray techniques is predicated on the absorption properties of the sample in question as dictated by the Beer–Lambert law, which can be given as

$$I_{t}=I_{0}e^{-\mu x}$$

where *I_t_* is the intensity of the attenuated X-ray beam, *I_0_* is the intensity of the irradiating X-ray beam, *µ* is a linear attenuation coefficient and *x* is the sample thickness. As intimated by the coefficients of this law, contrast in these images may be dictated by both the intrinsic composition of the material or its density. Absorption contrast represents perhaps the most conceptually straightforward means of harnessing X-rays for the purposes of imaging.

The distinction between standard radiography and CT lies in the relative configuration of the X-ray source, detector and sample. Where simple X-ray radiography uses a static colinear arrangement of the X-ray source, sample and detector, respectively, to collect a single two-dimensional radiograph or projection, CT involves rotating the sample, typically through 180 or 360°, relative to the radiation source and capturing multiple projections. These projections are then computationally reconstructed to generate a three-dimensional volumetric image comprised a series of two-dimensional real-space images representing cross-sections of the sample. Notably, the geometric arrangement and reconstruction principles of CT are not unique to X-ray techniques and may indeed be employed with a range of other radiation sources such as γ-rays [2], neutrons [3,4], electrons [5,6] and muons [7,8].

Since its inception and to this day, XR-CT is most popularly recognized as a medical imaging modality, being used in the early 1970s by Sir Godfrey Hounsfield (whose namesake is immortalized in the discipline as a quantitative scale for describing radiodensity) to perform the first live brain scan of a patient with a cerebral cyst [9], work that would ultimately culminate in the receipt of the 1979 Nobel Prize in Medicine [10]. Through the 1980s and 1990s, the use of XR-CT expanded into broader research communities. Initially adopted by clinically adjacent biomedical research communities [11], it was soon espoused by the physical sciences as a non-destructive testing (NDT) method for ceramics and engineering components [12–14]. This period also saw the first significant reductions in voxel size (i.e. the smallest addressable three-dimensional element in a volumetric image analogous to a pixel in two-dimensional images) and by extension improved resolution with the first recorded use of higher brilliance synchrotron radiation [15,16] and the commercial availability of more sophisticated acquisition and detection apparatus for laboratory-based CT set-ups. The reader is referred to an informative figure illustrating this reduction in voxel size in Scharf *et al*.'s recent review of XR-CT for battery research [17]. Increased focus on XR-CT for materials systems was seen through the 2000s with early tomograms of fuel cells [18] and batteries [19] and the addition of *in situ* and *operando* modalities from the early 2010s [17].

Contemporaneous to research addressing the desire for improved XR-CT resolution in the 1980s was work seeking to expand the quantitative possibilities of CT techniques. An early candidate selected to this end for translation into CT was X-ray diffraction (XRD), a phenomenon occurring during the coherent scattering of X-rays and already used widely at the time to determine atomic structure. The first reported example of laboratory-based XRD-CT can be attributed to Harding, Kosanetzky and Neitzel in their 1987 work using a pencil beam set-up to characterize plastic phantom samples [20], while the earliest example of synchrotron XRD-CT is not seen until almost a decade later in 1998 with Kleuker *et al*.'s feasibility study of a medically topical soft tissue sample at the medical imaging beamline of the European Synchrotron Radiation Facility (ESRF) [21]. Further technique development has seen the method being optimized for material samples with larger fields of view [22] and the addition of temporal resolution or environmental control, which are seen in studies referring to dynamic three-dimensional-XRD-CT or five-dimensional (e.g. three spatial, one diffraction and one temporal dimension) XDS-CT studies.

#### (ii) XRD-CT

**Figure 1.**
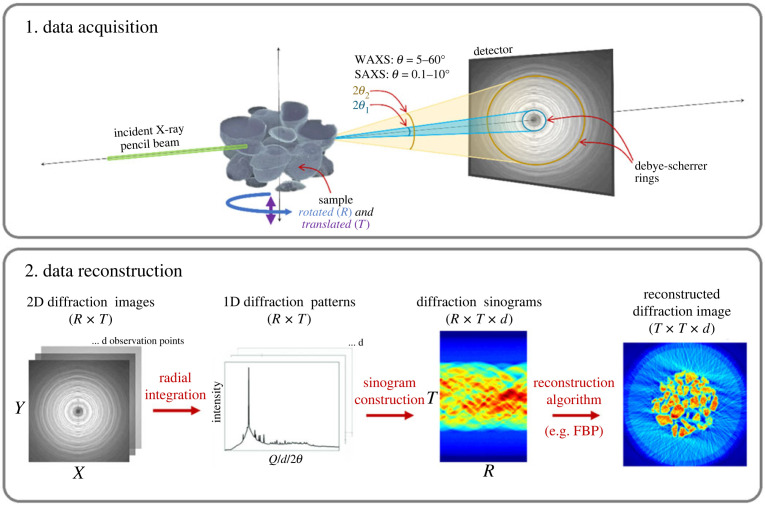
Summary of XRD-CT data acquisition and reconstruction. Note the following terms in the above for *R* (rotation), *T* (translation), *d* (observation points) and FBP (filtered back projection).

The principle of XRD-CT, also referred to as ‘diffraction tomography' or ‘diffraction imaging', is foundationally described by Harding *et al*. [20] and was first applied at synchrotrons in the late 2000s [23,24]. In XRD-CT, a sample is irradiated with a monochromatic X-ray pencil (i.e. highly collimated) beam, as summarized in figure 1. An area detector is then used in transmission geometry to collect two-dimensional powder diffraction patterns, although it is noted that this can also be achieved with a zero- or one-dimensional detector albeit with lengthy acquisition times. Some early efforts used a white beam and energy resolving detectors [25,26]. To achieve a volumetric image, the sample is both rotated through 180° about an axis oriented perpendicular to the incoming beam. By translating along this axis (originally step-wise but more recently with continuous rotation or fly-scanning [27,28]), it is also possible to obtain three-dimensional-XRD-CT data, which is simply a stack of tomographic cross-sections that are then computationally rendered together to achieve a full volume. For each translation step (*N_y_*), *N_ω_* diffraction patterns are collected for a series of rotational positions. A complete three-dimensional stack may be comprised of tens of thousands to millions of diffraction patterns. Integrating and averaging the azimuthal direction of the two-dimensional patterns produces intensity-based diffraction profiles.

Cross-sectional images are then created by reshaping the integrated data into sinograms and finally reconstructing the real-space images computationally, as summarized in figure 1. There exist a range of different reconstruction algorithms that can be classified into two main types: direct or analytical methods, where images are computed from the sinogram data using mathematical operations involving Fourier transforms (direct Fourier reconstruction) or simple back-painting combined with a filter; and iterative methods, which iteratively update the real-space images to converge towards sinogram data after forward projecting them. The most commonly used direct method is filtered back projection (FBP) while some popular iterative methods include algebraic reconstruction technique (ART) and the simultaneous iterative reconstruction technique (SIRT). The development of reconstruction techniques that address the specific needs are a topic of ongoing research, especially in cases where the sample or acquisition parameters are particularly demanding. Some examples include methods to address the parallax effect where the sample size is significantly large [29,30] and methods that work with limited datasets [31]. A third type of reconstruction method that has emerged out of these efforts are deep-learning algorithms, which are discussed further in §3. Post reconstruction, XRD-CT data can be analysed as conventional XRD using full profile analysis methods, such as the Pawley, LeBail and Rietveld methods [32,33]. Indeed, these have been combined with multivariate curve analysis to identify and map amorphous phases [22]. When powered by appropriate XRD data analysis software, like TOPAS, Rietveld refinement of 10 000s to 100 000s of diffraction patterns can be performed in several hours. However, as samples become more chemically complex and datasets become larger, using the mean diffraction pattern as a starting model for refinement can cause the process to become unstable. The time to analyse can become protracted where starting models need to be iterated multiple times. Similar to reconstruction, deep learning is also emerging as an alternative method for guiding refinement and achieving ultra-fast phase identification.

At this point, the difference between XRD-CT and the closely related techniques diffraction contrast tomography (DCT) [34–37] and 3DXRD [38,39] are explained, although as a relatively well-established technique developments in DCT are not highlighted as a focus of this review. XRD-CT is technically a powder diffraction technique, meaning that each pixel of a reconstructed image corresponds to a full one-dimensional powder diffraction pattern. Conversely, 3DXRD and DCT (using pencil beam and full-field approaches, respectively) use the isolated diffraction peaks (aka diffraction spots) generated by large single crystals to reconstruct grain maps, which may also contain quantitative information regarding strain and orientation. In both cases, if the sample being analysed begins to display diffraction features not suited to the technique (i.e. single crystal artefacts in the case of XRD-CT or powder diffraction-like spot overlap in the case of DCT/3D-XRD), reconstruction and data analysis becomes exceedingly difficult and may require the application of filters to render the data usable [40]. Unlike XRD-CT, DCT can be more readily performed in the laboratory with the commercial release of Zeiss' laboratory DCT [41].

#### (iii) SAXS-CT

The term ‘XRD’ is often used interchangeably with the more specific term wide-angle X-ray scattering (WAXS) to describe a diffraction technique analysing X-rays scattered between 5–60°. By contrast, SAXS analyses elastic scattering at small angles (i.e. 2*θ* ≈ 0.1–10°) (figure 1). By Bragg's Law, the two angle ranges correspond to two different length scales, with WAXS resolving sub-nanometre (i.e. 1–0.1 nm) structures and SAXS resolving nanoscale (i.e. 1–100 nm) structures. WAXS is thus used to characterize the d-spacing of crystalline materials while SAXS can be used to characterize samples with larger-scale structure such as colloids [42], nanoparticles [43], proteins [44], DNA assemblies [45], soft tissue [46] and bone [47–50] among others.

The primary difference between a WAXS and SAXS measurement is the distance from the sample to the detector, which is shorter in WAXS. This means it is not uncommon to find modern set-ups that can acquire both WAXS and SAXS, sometimes even simultaneously, providing full-scale characterization of hierarchical materials. Like XRD-CT, combining SAXS with CT enables variation in nanostructure to be spatially resolved over a large field of view. This is of particular interest for anisotropic structures like polymers, glassy materials and biomaterials.

While early examples of SAXS-CT date back to the 2000s [51], it has only recently become more widely used as reconstruction is more challenging than in XRD-CT. Typically, reconstruction techniques hinge upon the signal acquired being rotationally invariant (i.e. the sum of line integrals forming a projection are independent of the sample rotation), which is not necessarily the case in SAXS-CT data where samples of interest are more likely to be highly anisotropic [52]. As such, early SAXS-CT studies were restricted to materials with preferential orientations (e.g. isotropically scattering powders, aligned collagen), undermining the true potential of the technique. More recently, alternative reconstruction methods have been successful in preserving oriented SAXS signals, enabling SAXS-CT to be performed on more complex samples [52,53].

#### (iv) XRD-TT

XRD tensor tomography (XRD-TT) is a derivative of XRD-CT and SAXS-CT that reconstructs the scattering tensor for each voxel. Ideally, XRD-TT data should be acquired on a set up with two angular degrees of freedom to obtain projections through multiple tilted axes. The purpose of this is to address signal arising from anisotropic scattering, which is dependent on the orientation between the sample and the beam, and to achieve higher quality reconstructions. XRD-TT data acquisition is inherently longer as a result and a handful of studies have attempted to address this with alternative acquisition geometries [54]. In recent times, the use of circular grating arrays have enabled faster acquisition [55].

#### (v) PDF-CT

A further scattering variant is PDF, a function that expresses the probability of encountering atom pairs separated by a given distance. PDF data is acquired in much the same way as XRD data but probes total scattering (i.e. Bragg and diffuse) over a larger range of reciprocal space, which is expressed in terms of the scattering vector Q. In PDF measurements, *Q* is much higher than in standard measurements (i.e. ≈30 versus 5 Å^−1^ in PDF versus XRD) [56]. A one-dimensional PDF is then obtained by applying a Fourier transform after a normalization of the one-dimensional diffraction patterns has been performed. The result is a method that provides information on nanostructured materials. The more complete history of PDF is covered in a recent review by Billinge [56].

Synchrotron PDF-CT studies have emerged in the last decade as a method of mapping quantitative nanostructure parameters. Jacques *et al*. demonstrated the initial feasibility of PDF-CT in 2013 on a series of amorphous and semi-crystalline phantom samples as well as an *in situ* model of an industrial Pd catalyst by comparing results to standard XRD-CT [57]. Early unsuccessful attempts to demonstrate PDF-CT on a battery sample with strongly scattering components highlighted the need for statistically significant data volumes when attempting to obtain PDF as well as PDF-CT-specific reconstruction algorithms in the future [58].

#### (vi) X-ray-Spectroscopy-CT and hybrid methods

Although beyond the scope of this review, X-ray spectroscopy is briefly mentioned in its capacity as an adjunct to XDS-CT methods. Spectroscopic techniques like XRF, energy-dispersive X-ray spectroscopy (EDX), and X-ray absorption spectroscopy including X-ray absorption near edge structure (XANES) can often be performed either alongside or simultaneously with XDS-CT methods with the use of multiple offset detectors. Early examples of combined micro-XRF/XRD-CT date back to the mid-2000s [59]. Combined studies expand the depth of information obtained and can be used to corroborate each other's results. The use of correlative imaging experiments (i.e. same sample measured on a different set-up) have also been piloted, such as an XRD/neutron-CT experiment performed at DESY and FRM II [60]. Such studies attempt to maximize the potential interpretation of results by exploiting the varied means by which contrast is generated.

### (b) Current capabilities

#### (i) Synchrotrons

XDS-CT requires the use of a pencil beam and is thus performed almost exclusively at synchrotrons because they are the only sources that can provide stable high-energy X-rays (i.e. 30–60 keV) with nanofocused collimation (i.e. 150–500 nm), high brilliance (greater than 10^18^ photons s^−1^ mm^−2^ mrad^−2^/0.1%BW) and low emittance [61–63]. The brilliance of a synchrotron is unparalleled by any laboratory-based source, being some 20 orders of magnitude greater than standard X-ray tubes [64]. High flux is desirable in XDS-CT to reduce measurement times, which can be long due to the high number of angular projections and lateral translations required to build a full tomographic set. Indeed, the current experimental bottleneck with synchrotron XDS-CT is no longer the ability to collect sufficient data but rather data processing methods that can contend with the extremely high volumes of data that can now be produced.

Worldwide there are over 50 different synchrotrons with beamlines focusing on X-ray nanoprobe imaging now routinely offering resolutions well below the optical diffraction limit. While this review does not necessarily capture all operational synchrotrons with up-to-date XDS-CT capabilities, it does include studies from synchrotrons in the UK (i.e. diamond light source (DLS)), Europe (i.e. European Synchrotron Radiation Facility (ESRF), Deutsches Elektronen-Synchrotron (DESY), Swiss Light Source (SLS), Source optimisée de lumière d’énergie intermédiaire du LURE (SOLEIL)), Japan (i.e. SPring-8), Brazil (i.e. Brazilian Synchrotron Light Laboratory (LNLS)), America (The Advanced Photon Source (APS), Cornell High Energy Synchrotron Source (CHESS)) [65] and China (Shanghai Synchrotron Radiation Facility (SSRF)) [66]. Of these, attention is drawn to the beamlines ID15A [67], which features in a number of the highlighted studies, ID 11 [61], ID13 & ID31 at ESRF, P07 at DESY, and 1-ID at APS [68]. A practical limitation and potential deterrent of synchrotrons is limited accessibility, which typically sees researchers lodging applications for high-demand beamlines months in advance in hopes of being allocated facility time.

#### (ii) Laboratory

The limited accessibility of synchrotrons means there remains a sizable market for lab-based XDS-CT equipment. A commercialized example is Zeiss' LabDCT, a lab-based adaptation of DCT that can provide non-destructive three-dimensional grain mapping of materials samples using a polychromatic conical beam. However, there are still intrinsic challenges associated with the low flux of in-house X-ray sources. While synchrotron XDS-CT uses a pencil beam, such low utilization efficiency of a low-brilliance source results in impractically protracted acquisition times and higher radiation doses for the sample. Some innovative solutions have been proposed and used including the use of alternative beam shapes (e.g. a coded cone-beam [69]) as well as alternative sinogram reconstruction methods, which include algorithms optimized for alternative beam shapes [30] and for datasets with limited projections angles [70]. Despite this, the data quality produced by such set-ups remains non-ideal as evidenced by recent studies proposing further reconstruction algorithms for already commercialized DCT units [71].

An emerging variant showing promise in laboratory set-ups is dark-field CT, which uses a grating interferometer to exploit ultra-small angle X-ray scattering signals. Originally piloted at synchrotrons, it has recently been translated successfully in the medical imaging field and scaled up to the human scale [72–76], although it is not yet clinically in use. While it is possible to perform quantitative analysis with dark-field set-ups, the resolution is not yet comparable to synchrotron XRD-CT and remains an area of ongoing development.

## 2. Recent insights from XDS-CT

### (a) Inorganic materials

#### (i) Materials science

Materials science is an interdisciplinary field that uses characterization to explore the relationship between structure, properties, processing and performance. Historically rooted in metallurgy and mineralogy, the field has now vastly expanded to include polymers, ceramics, composites, functional materials and biomaterials (discussed further in §2b). It is well established that heterogeneities in the microstructure of materials can have a significant impact on the mechanical, electromagnetic, thermal and optical properties of the material. Such heterogeneities include point defects like vacancies or interstitials, line defects like dislocations, planar defects like stacking faults or grain boundaries, and macroscopic defects like phase variations, cracks and precipitates. The development of advanced imaging methods has proven indispensable in demonstrating the manner in and extent to which spatial distributions of heterogeneities correlate with altered material properties.

DS analysis methods like powder XRD are mainstay characterization methods in materials characterization. It follows then that XDS-CT have been readily adopted by materials researchers as they can provide quantitative maps of structural features that might directly influence the sample's properties. Metallic samples in particular have always benefitted from DS analysis; even beyond being primarily defined by their structural parameters, the macroscopic properties of metals are largely attributed to the nature of their microstructure including the morphology of their grain boundaries and the interaction of structural defects (e.g. dislocations). Tomographic imaging has been performed on all manner of metallic samples from single crystals to granular microstructures to nanoparticles, with metallic samples often being used as benchmarks during XDS-CT technique development owing to the clarity of signal that can be obtained and this strong structure–function relationship.

##### XRD-CT

The ability for XDS-CT to elucidate static structure in metal structures is already well established. In recent years, trends in XDS-CT studies of metal samples have moved towards *in situ* studies evaluating the impact of strain or thermal treatments on structural and mechanical changes. A topic of perennial interest for metallurgists, the study of steel phase transitions has benefitted of late from the application of XRD-CT. Toda *et al*. [77] multimodally assessed mechanically-induced austenitic 0.1C-5Mn-1Si multi-phase steel transformation during tensile deformation at SPring-8's BL20XU beamline. In combination with high-energy X-ray nano-tomography, XRD-CT data revealed individual transformation behaviours dependent on the initial orientation and size of grains with greater stability recorded for coarser austenite grains than previously understood from bulk characterization techniques. The impact of interaction with soft and hard phases was also noted, demonstrating XRD-CT's utility in unravelling the microstructural contribution to phase transformations in steel. Similarly, Sedlak *et al*. used a tension-twisting rig at ID15A, ESRF to spatially evaluate austenite–martensite transition zones in an NiTi wire during tension and twisting [78]. Highly heterogeneous, distinct phase gradients were observed both in and beyond the transition zone. The localized effects observed are in contrast to what is typically extrapolated from macroscopic stress–strain curves, highlighting the value of a real-time volumetric imaging modality.

XDS-CT has also proved useful in analysing complex composite materials like cement. three-dimensional XRD-CT was used to observe the mechanical behaviour of non-cemented and lightly cemented quartz particles under *in situ* uniaxial compaction at F2, CHESS [65]. Seven load steps were applied in the beamline with a three-dimensional XRD-CT dataset being acquired each time. Particle fragmentation and contact particle kinematics were tracked with the cemented sample showing less fragmentation and less particle movement. The cementation network was found to distribute stress concentration at particle contacts, reducing the risk of fracture and enhancing coaxiality between principal stresses and strain.

##### XRD-TT

Analysis of XRD profiles with *a priori* information can provide a wealth of implicit structural information beyond simply phase identification based on d-spacings, including lattice strain and orientation. XRD-TT is a useful method for evaluating the scattering tensor of a sample's microstructure. Mürer *et al*. studied Pierre shale using XRD-TT to show the nanostructure orientation of clay minerals at ID15A, ESRF [79]. Shale is comprised of various mineral phases with different shaped flakes and grains that give rise to anisotropic small- and wide-angle scattering. In combination with XRD-CT and attenuation-contrast CT, clastic inclusions filled with multi-granular clinochlore and pyrite crystallites were also visualized in the shale sample.

##### SAXS-CT

Ordered non-crystalline samples can now be analysed using SAXS-CT. A popular example of such a sample are polymers, which lack the fine crystallinity of an alloy, for example, but still have some nanometric ordering with the alignment of their constituent monomer units. Injection moulded polymers will often exhibit a hierarchical semi-crystalline structure that evolves over time from when they are cast as liquids and set to solids. Hu *et al*. used SAXS-CT to show the spatial distribution of ordered lamellae in injection-moulded polylactide with different shear durations that are apparently influenced by shear flow during injection [80]. Meng *et al*. also used SAXS-CT to monitor the microstructure of injection-moulded poly (butylene adipate-co-terephthalate) (PBAT), also finding that secondary flow during the packing stage of the process led to an internal vortex ring that had not been well documented in previous studies [81]. In both cases, the use of SAXS-CT helps to elucidate the impact of different processing conditions on the microstructure of the final polymeric material.

#### (ii) Batteries

**Figure 2.**
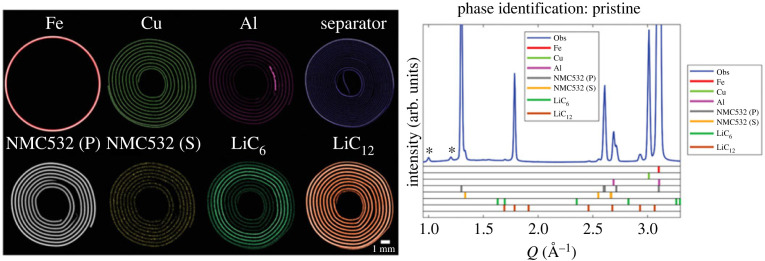
XRD-CT exemplar in Li-ion batteries showing spatial distribution of all components in a pristine NMC532 AAA battery cell where (P) and (S) indicate the primary and secondary phase components. Reproduced from Vamvakeros *et al*. [82].

Decarbonization of energy sources has led to unprecedented demand for high-performance batteries, especially in automobiles and portable electronic devices. The diminutive scale of many battery systems makes them a superlative pairing for the resolution offered by X-ray CT studies. Absorption-contrast X-ray CT has been used to generate some compelling images of the internal structure of batteries over the decades [17], which have been consolidated in recent times by advanced *in situ* XDS-CT studies that employ an engaging use of colour to spatially represent structural changes and the evolving distribution of chemical intermediates (see figure 2). The impact of synchrotron beam damage, which appears to significantly attenuate the behaviour of reaction mechanisms especially during operation, should be duly noted here and considered more routinely in future studies [83,84]. In particular, two distinct lithiation mechanisms in *operando* NMC battery studies have been tentatively associated with radiation dose, highlighting the need for studies using systematic dose-rate metrics on different battery systems to better establish margins of error in synchrotron studies.

##### XRD-CT

XRD-CT has proved a useful tool in providing evidence of chemical heterogeneities and crystallographic changes, often cycling-induced, in NCA [85], NMC [82,86], LMO [87], LFP [88–90], graphite [91,92], Na-based [93] and Li-S [94] batteries. Some studies provide evidence of strain introduced during cycling that could impact the overall mechanical integrity and lifetime of the battery. For example, during cycling of an NMC battery at 4.2 and 4.7 V, Daemi *et al*. demonstrated a voltage-dependent shrinkage of electrodes and a high heterogeneity of internal structures that also experienced cracking during cycling [86]. Elia *et al*. also observed an increase in strain with cycling at the PG electrode of an aluminium/graphite battery that led to irreversible volumetric changes [91].

Heterogeneity of reactions across electrodes was studied in an LFP battery by Liu *et al*. who found accelerated reactions at the electrode faces that had contact with the battery separator or current collector and were able quantify a relationship between non-uniformity and variability in discharge rates [88]. Similar heterogeneity was observed by Tonin *et al*. in a dedicated Li-S battery cell designed for more accurate tomographic *operando* studies of electrodes [94]. A study of an NCA also found different degrees of lithiation at the anode during charging, with some regions exhibiting homogeneous lithiation, and others exhibiting variably delayed lithiation [85]. Similar to Liu *et al*., contact with various structural components of the battery like the Al tab led to variation in charging behaviour and lattice parameters. Battery XRD-CT studies are perhaps notable for the relative consistency in cycling trends emerging across different studies even for dissimilar battery systems.

##### PDF-CT

PDF-CT was combined with *operando* techniques in two studies by Sottmann *et al*. on Li- and Na-ion batteries [95,96]. The first focused on the cycling of sodium in a phosphorous anode using a working cell that had not been significantly modified, representing one of the better XDS-CT studies on a real-world battery system. Their follow-up study used fie dimensions (where the fourth and fifth dimensions are the time and chemical dimensions, respectively, with the latter being derived from analysis of the diffraction pattern) PDF-CT of a bismuth vanadate Li-ion battery anode, demonstrating a combined conversion/alloying mechanism of Li-Bi alloy at the anode, which were protected by an amorphous matrix of lithium vanadate. In both cases, PDF was used to study materials of interest that transitioned through an amorphous phase during battery cycling, which marks an improvement on XRD-CT studies that can only provide chemical information on the crystalline phases present.

#### (iii) Fuel cells

Fuel cells are electrochemical cells that produce electricity via redox reactions. Typically classified by their electrolyte (with polymer electrolyte membrane (PEM), direct methanol, alkaline and solid oxide fuel cells as examples), they can also be considered in terms of the temperature domains within which they operate (i.e. high and low). Over the last few decades, research into fuel cells has been approached with a sense of optimistic ambivalence. On the one hand, fuel cells have the green potential of significantly lower (or in the case of hydrogen cells, no) emissions and higher efficiency than combustion engines; on the other, practical efforts to date have been unsuccessful in commercializing many proposed fuel cell designs.

Studying fuel cells in synchrotron beamlines can be challenging as the layered structure of cells can make it difficult to isolate regions of interest such as electrodes or water transport mechanisms [97]. Additionally, it is known that the performance of fuel cells degrades rapidly when exposed to high-energy synchrotron radiation, which has hampered the progress of *in situ* studies [97–99]. The requirements for studying fuel cells is indeed complex enough to have warranted the construction of a dedicated beamline at SPring-8 for the purposes of studying polymer electrolyte fuel cells (PEFCs) with specifications designed to facilitate simultaneous *in situ* XAFS/XRD-CT, as well as QXAFS and HAXPES for PEFCs [100]. Some studies isolate materials used in the fuel cell and either perform static XDS-CT or observe changes under simulated conditions. For example, Heenan *et al*. used three-dimensional XRD-CT to observe changes in a re-oxidized Ni-YSZ solid oxide anode at operational temperatures (i.e. >800°C) and were able to visualize non-isotropic lattice distributions, metallic strain, and evidence of monoclinc zirconia at oxidation boundaries, all of which serve to characterize the electrode's mechanical integrity and can be used to infer the component's lifetime in a real cell [101].

Other work for fuel cells involves developing designs that enable tomography studies. For example, Martens *et al*. reported a design for an X-ray transparent proton exchange membrane hydrogen fuel cell using a grazing incidence geometry to increase the signal-to-background ratio while still accurately reproducing the electrochemical performance of a standard device [102]. The fragility and poor performance of small solid oxide fuel cells was addressed in an *in situ* XRD-CT at ID15A, ESRF piloting the performance of a ‘micro-monolithic' ceramic fuel cell under real-life conditions [103]. The design was based on ceramic honeycomb monoliths used in existing catalytic applications that offered high strength-to-weight ratios and excellent shear strength. Compared with standard fuel cells with a conventional microtubular geometry, the monolith design showed superior stability during thermal cycling while achieving a power density of 1.27 W cm^−2^ at 800°C as well as excellent fracture resistance during bending. Regarding the degradation of Pt catalysts, *operando* XRD-CT also revealed that heterogenous degradation gradients across the catalyst were associated with water distribution and flow through the cell [104].

#### (iv) Catalysis

Catalysis is a multi-million dollar industry concerned with the use of specialist catalytic materials to increase the rate of chemical reactions. Used ubiquitously throughout chemical industry, catalysis has been a key element in enabling the commercial production of a cornucopia of different substances including fuels, fertilizers, cleaning agents and pharmaceuticals. Traditionally used to improve the economic efficiency of chemical production workflows, a collective global focus on sustainability has seen reprioritization of catalytic processes that can reduce industrial environmental footprints [105] (e.g. decarbonization and emissions control technologies [106]). Some examples of topical catalytic systems in research today include fluid catalytic cracking (FCC) [107], methanol-to-hydrocarbon (MTH) reactions [108], methane dehydroaromatization [109], propane dehydrogenation [110], biomass conversion [111] and Fischer–Tropsch synthesis (FTS) [112]. The chemical complexity of catalytic reactions is reflected in the broad array of research interests within the field, which range from fundamental mechanistic work seeking to consolidate a first-principles model of catalytic processes to catalyst design to end-stage process integration and optimization.

In recent times, some of the greatest novel catalytic insight can be attributed to the development of microscopic techniques. Previously only understood in terms of their bulk performance, microscopy has vastly increased the current understanding of catalytic materials by spatially mapping their structure–function relationship. XDS-CT methods are of particular interest, as they allow visualization of a bulk catalyst's internal structure under operational conditions. Some recent contributions are summarized here.

##### XRD-CT

A considerable number of *in situ* catalytic XRD-CT studies have been performed at beamlines ID11, ID15A and ID31 of the ESRF, many by the Beale group, on aspects of oxidative coupling of methane (OCM) [113–117], partial oxidation (POX) of methane [28,118], CO_2_ methanation [119], the low-temperature water–gas shift [120] and gas separation catalysts [121]. The catalytic conundrum that is coking has also been studied [122]. OCM is a reaction for the conversion of natural gas (i.e. methane) into ethylene, one of the most important commodity chemicals. Commercialization of OCM is prohibited by its non-economically viable yields, which can be attributed to poor catalytic performance (including activity, selectivity and stability), propensity for unwanted CO_x_ formation via surface adsorption of oxygen, and premature methane combustion [123].

XRD-CT studies of fixed bed La-Sr/CaO OCM catalysts with low ethylene yields (due to high-temperature formation of CO2 and CO) have previously revealed temperature gradients across the powder beds attributed to the presence of various SrCO_3_ polymorphs that promote different forms of oxidation across the bed [117]. The stability of SrCO_3_ was found to be dependent on ambient gas conditions (e.g. pressure, flow) and prone to additional degradation pathways in a further XRD-CT study [115], demonstrating the capacity for packed beds to become increasingly heterogeneous throughout operation. Membrane reactors, which supposedly offer improved C_2_ selectivity in OCM by controlling the quantity of available oxygen to react, have also been evaluated in XRD-CT studies that provide clear volume rendering of chemical evolution under operational conditions. Two BCFZ (2% Mn-1.6% Na-3.1% W/SiO_2_; 2% La-2% Mn-1.6% Na-3.1% W/SiO_2_) and an LSCF (2% La-2% Mn-1.6% Na-3.1% W/SiO_2_) membrane reactors were studied [114] under operating conditions as well as a Mn-Na-W/SiO_2_ catalyst [124]. Both studies were able to propose improved formulations, BaCo_0.4_Fe_0.4_Zr_0.2_O_3−δ_ and La-promoted Mn-Na-W/SiO_2_, respectively, based on the performance observed *in situ*. A BSCF (Ba_0.5_Sr_0.5_Co_0.8_Fe_0.2_O_3−δ_) hollow fibre membrane and Na–Mn–W/SiO_2_ catalyst were also compared *in situ*, with XRD-CT data demonstrating the structural collapse of the primary BSCF phase followed by the formation of secondary phases at high temperatures, providing mechanistic insight into the medium- and long-term stability of this catalyst formulation [113].

Catalysts used in the associated POX reaction have been studied, illustrating a spatial dependency for the formation of unstable Ni-Pd alloys, coke deposition and Ni sintering [28]. XRD-CT has also been used as part of a suite of multimodal characterization studies for a series of three-dimensional printed catalysts for use in OCM [116], gas separation [121] and CO_2_ methanation [119], providing information about the spatial distribution of catalyst and assessing the fidelity of three-dimensional printing.

Nickel poisoning, a major cause of catalyst deactivation, of an FCC catalyst was studied by Gambino *et al*. in a multimodal µXRF-µXRD-µXANES-CT experiment at X05LA, SLS [125]. Tomographic data spatially resolving Ni interaction across the catalyst found preferential interactions with the catalyst's γ-Al2O3 matrix, leading to the formation of Ni-enriched spinel hotspots that can act as nucleation spots for coke accumulation and hydrothermal degradation. XRD-CT was used to identify the various phases of alumina and Ni-rich spinels as well as characterizing the nature of hydrothermal damage by tracking changes in lattice parameters.

##### PDF-CT

**Figure 3.**
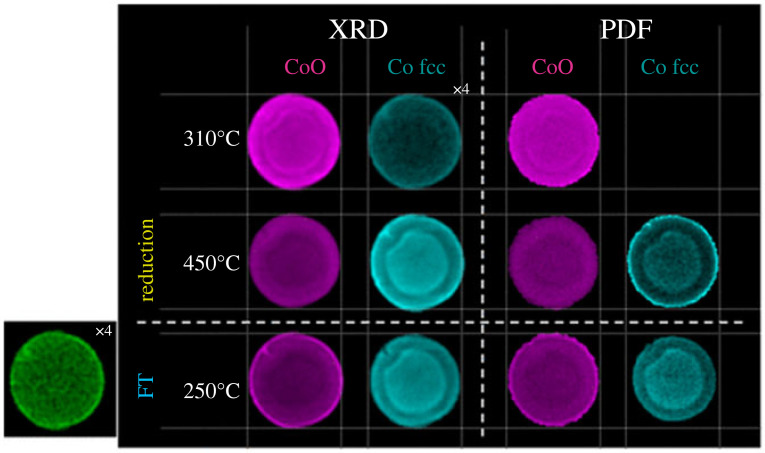
PDF-CT exemplar in Fischer–Tropsch catalyst. Reconstructed two-dimensional integrated Fourier transform intensity maps based on the intensity of the Co–Co scattering features at approximately 3 Å (CoO) and 2.5 Å fcc Co are shown under reduction and during FT conditions as a function of temperature and time. Note corresponding XRD-CT data shown in the left panel. Reproduced from Senecal *et al*. [126].

PDF-CT was applied in conjunction with XRD-CT at ID15A, ESRF to study a Co/γ-Al_2_O_3_ during the early stages of FTS [126], as shown in figure 3. PDF-CT was selected as a complement to better visualize small nanocrystallites (less than or equal to 6.5 nm) known to exist in the sample that yield lower signal intensity with standard XRD-CT. Two types of Co3O4 particles were seen: those well dispersed interacting strongly with the alumina matrix and poorly dispersed aggregated particles exhibiting lower matrix interaction. Smaller, strongly bound particles resolved in PDF-CT appear to remain as CoO during FTS treatment rather than being fully reduced while loosely bound and larger particles found towards the edge and very centre of the pellet appear to reduce more rapidly, illustrating the challenge in synthesizing homogeneously active catalysts, even on a small scale. The combination of XRD and PDF-CT enables all microstructural length scales to be appropriately analysed.

#### (v) Cultural heritage

The ‘non-destructive’ aspect of NDT is imparted with a heightened sense of gravity in the context of cultural heritage, a field that seeks to use analytical techniques to direct preservation strategies for tangible cultural property. Cultural heritage samples are typically delicate, prone to damage and irreplaceable, which collectively translates to a need for extremely lowly (if at all) destructive analytical methods. Unsurprisingly, characterization techniques used by conservation scientists are (for want of a better word) conservative and include XRF [127] and visible light spectroscopy (primarily photoluminescence [128] but also Raman [129]) with an interest in portable devices for minimally disruptive *in situ* measurement [130]. Priorities are generally centred around understanding the evolving surface chemistry of cultural items with the view to understand how best to store [131], clean [132] or maintain them. Analytical methods also provide a quantitative complement to the efforts of historians seeking to more accurately document artistic practices throughout time [133]. A small but promising handful of studies have begun demonstrated the benefit of synchrotron-based XDS-CT methods in conservation science.

##### XRD-CT

Unidentified deposits and precipitates in paintings pose a challenge to conservation strategies. While XRF and XAS mapping are popular techniques for conservation [134] they can struggle to discriminate between closely related crystalline compounds and provide an incomplete picture of evolving deposit chemistry. The development of microscopic and macroscopic two-dimensional XRPD mapping methods has provided superior chemical identification but remains unable to provide organized depth information pertaining to the layers of paint applied.

**Figure 4.**
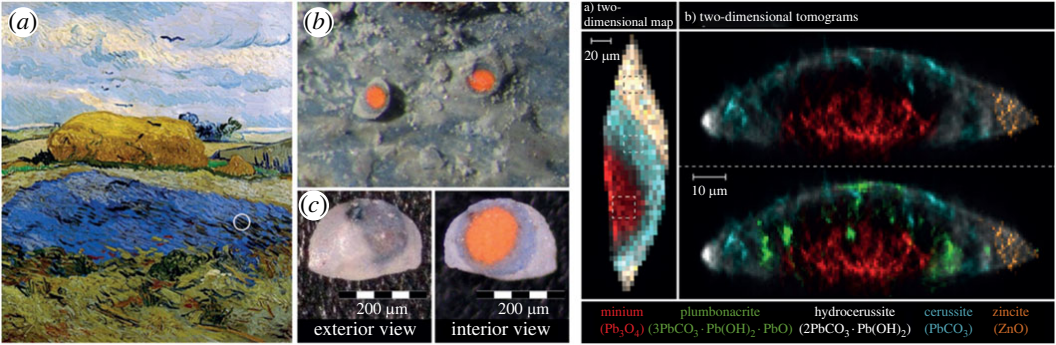
XRD-CT exemplar from a microparticle of van Gogh's Wheat Stack Under a Cloudy Sky (1889) (left) (*a*) Photograph of Wheat Stack Under a Cloudy Sky by Van Gogh (October 1889, oil on canvas, Kröller-Müller Museum, NL) with white circle denoting sample area and *b*/*c*) detail of paint sample. (Right) XRD-CT reconstructions of (*a*) projected and (*b*) internal crystalline distribution of the paint sample. Reproduced from Vanmeert *et al*. with permission from John Wiley & Sons [135].

One of the first cultural heritage applications of XRD-CT was performed at P06 PETRA III, DESY on a micro-particle from van Gogh's *Wheat Stack Under a Cloudy Sky* (1889) to understand the causes of red Pb pigment discoloration [135], shown in figure 4. The presence of plumbonacrite, a rare lead carbonate with minimal documentation in pre-twentieth century paintings, was reported providing more detailed insight into photoinduced lead oxide degradation processes.

More recently, XRD-CT was used to investigate Pb-rich deposits emerging on the surface of *Homer*, a 1663 oil-on-canvas by Rembrandt van Rijn and an excellent example of both the searching, introspective portraiture that characterized his later years as well as his experimental paint formulations that featured smalt and earth pigments. XRD-CT of a paint micro-sample at I18, DLS revealed a surface crust of lead sulfates including palmierite, anglesite, lanarkite, leadhillite and (hydro)cerussite atop a base of calcite and earth pigment [136]. In particular, XRD-CT spatially resolves a decreasing S:Pb ratio from the surface of the painting, enabling the proposal of a deposit formation mechanism that includes Pb migrating upwards through the paint layers via ‘ion hopping' over the carboxylic acid into the upper layers where they preferentially react with external sources of S (likely SO_2_) to form sulfates. The same method at I18, DLS was also applied to a further three Dutch Old Master paintings revealing a similar build-up of palmierite, anglesite, and lanarkite also associated with environmental SO_2_, previous restoration efforts, and stratigraphic paint build-up [137]. Importantly, in both cases XRD-CT indicates that deposit removal would not be possible without damaging the original layer and provides insight into potential long-term environmentally controlled storage strategies for oil paintings.

XRD-CT has also recently been applied to fifteenth-century French brocade samples incorporating gold and silver yarn [138]. As it was known the samples contained a range of unidentified phases, the study uses supervised multivariate curve resolution—alternating least-squares (MCR-ALS) analysis to automatically decompose data for more efficient phase identification. Tomography revealed that the brocades had been subject to various foiling, overpainting, and glazing techniques over time, which appeared to have then experienced several different degradation pathways. Similarly, an architectural heritage sample of stone was analysed in a proof-of-concept synchrotron XRD-CT study, which showed the distribution of calcium phosphates including hydroxyapatite (HAp) and octacalcium phosphate in a diammonium hydrogen phosphate sample, providing some cursory insight into potentially relevant conservation treatments [139].

##### PDF-CT

Synchrotron PDF-CT was used to characterize wood samples taken from the *Mary Rose*, Henry VIII's flagship navy carrack that sank in 1545 and was raised in 1982 [140]. Total scattering methods like PDF are useful for analysing materials lacking convincing long-range order but still potentially containing local nanostructures and can succeed where conventional XRD-CT struggles to acquire sufficient signal. In its history of conservation, wood from the *Mary Rose* has been coated with solutions of polyethylene glycol (PEG), which have also inadvertently led to unexpected conservation challenges with the formation of acid from PEG breakdown and PEG-induced creep both compromising its mechanical stability. The presence of amorphous or nanoscale guest PEG species in the waterlogged wood makes the sample a reasonable candidate for PDF-CT. A 5 nm zinc sulfide nanoparticles deposited by bacteria under anaerobic conditions were successfully identified during experiments on keelson wood at ID15A, ESRF and were further identified as precursors to acid attack following exposure to aerobic environments, informing future conservation strategies.

### (b) Biomaterials

Absorption-based XR-CT is a gold standard medical imaging technique that has been adopted worldwide. CT radiography offers a truer geometric representation of its subject than traditional planar radiographic projections do, which has made the method indispensable in scenarios where accurate visualization is necessary for delivering optimal clinical outcomes such as surgical planning, in particular orthopaedics [141] and neurosurgery. The early intentions to develop a quantitative form of XRD-CT for diagnostic radiology are evidenced in Harding, Kosanetzy and Neitzel's original paper on the method, which expressly illustrated the dissimilarity between fat, muscle, and bone XRD signals to demonstrate its eligibility for diffraction-based imaging [20]. However, XRD-CT's potential in the medical imaging space has to date been almost completely eclipsed by magnetic resonance imaging (MRI), which offers superior visualization of soft tissue structures, and its derivates functional MRI (fMRI) and diffusion-weighted MRI (DW-MRI), both of which have been instrumental in cementing MRI's status as a powerhouse, functionalized medical imaging modality capable of providing real-time feedback. By comparison, clinical translation of XDS-CT methods have veritably lagged, struggling to advance beyond the starting line first drawn by Harding *et al*. in 1987; at present there is no clinical XDS-CT instrumentation that can perform *in vivo* imaging on human subjects. Despite this, there is still scope for XDS-CT methods to be developed into commercial medical imaging units. Currently available absorption-contrast CT scans are both faster (c. 10 min for CT versus 45–60 min for MRI) and cheaper than MRIs, and X-ray methods are still the preferred option for visualizing bone and vasculature (albeit to a lesser extent). MRI is also contraindicated for specific patient groups (e.g. patients with implants or medical devices) and can be less tolerable for those prone to claustrophobia.

The prelude to any such clinical application of XDS-CT techniques is thus the demonstration of its feasibility in imaging mammalian tissue and biomaterials, a term that is broadly defined as a material (either naturally occurring or synthetic) engineered to interact with biological systems in a therapeutic or diagnostic manner. Biomaterials with an inorganic component such as bone and dentin [142] have been favoured in proof-of-concept studies, but successful experiments have also been performed in soft tissue. It is argued that the lack of broader medical interest is partially attributed to the fact that despite frequently being touted as a non-destructive three-dimensional imaging method, XDS-CT methods are not truly non-destructive in the case of biological samples, which are generally subject to excision and extensive sample preparation prior to imaging. To date, the majority of XDS-CT studies focus on technique validation for a particular class of samples. Uptake of DS-CT in biomedical communities will likely benefit from studies that move beyond proof-of-concept and instead demonstrate novel biomedical insight, with some examples including mechanistic understanding of disease processes or preliminary intervention studies. The reader is also referred to some recent reviews of DS-imaging of biological samples that are not limited to CT [143].

#### (i) Bone

Bone is a structurally complex naturally occurring, hierarchical composite material that is well-suited to XDS-CT imaging due to its high relative proportion of crystalline components, namely HAp and ordered collagen [144]. DCT has been useful here in *ex vivo* studies of bone and their interaction with implants [145] owing to its capacity to traverse larger areas of sample, which is useful in more clinically oriented studies. XDS-CT studies have been useful in visualizing the orientation of lamellar structure. It is noted among the studies reviewed that there is a lack of work on diseased bone (e.g. osteoporosis, arthritis, osteonecrosis, Paget's), which potentially limits clinical interest.

##### XRD-CT

In seeking to demonstrate the capabilities of XRD-CT on bone, fossilized specimens are a convenient option, featuring not only Hap but a variety of other XRD-resolvable mineral compounds arising as a consequence of fossilization. Having long-since met their demise, the need for ethical involvement in obtaining samples is also negated. Fossilized bones over 300 million years old from a tetrapod (*Discosauriscus austriacus*) and a lobe-finned-fish (*Eusthenopteron foordi*) were interrogated with an 86.6 keV beam at ID15A, ESRF to study Hap crystallite orientation, showcasing the technique's capacity to both identify and quantitatively describe unit-cell parameters [146]. A single tomographic slice was obtained for the tetrapod sample, while a full three-dimensional volume was rendered for the lobe-finned-fish sample with a lower resolution to cover a larger area of sample, which both revealed a preferential orientation for the unit cell c-axis of Hap crystallites parallel to the bone surface, allowing palaeobiological inferences to be made pertaining to, for example, the animals' bone growth or adaption to loads. Further, the ability to spatially identify various mineral phases (e.g. calcite, quartz) provides information on diagenesis. The utility of XRD-CT in unveiling strain directionality in biogenic crystals is also recently demonstrated in a comparable work on a non-osseous Mg-calcite material at ID16B, ESRF in a study showing extremely high internal strains in the single crystal dorsal arm plates of the brittle star *Ophiocoma Wendtii* [147].

**Figure 5.**
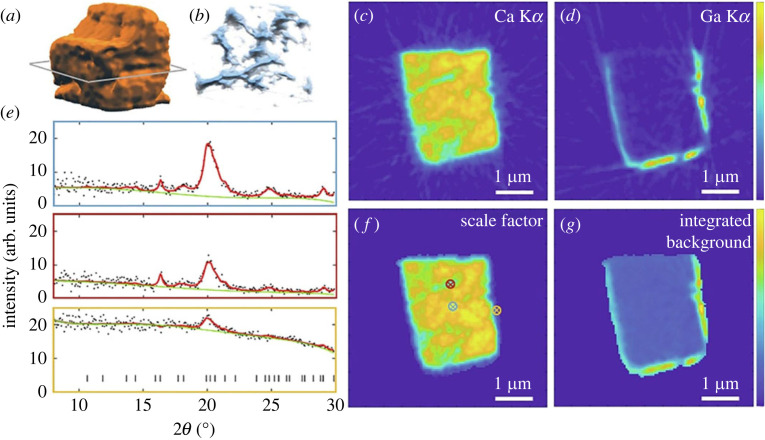
XRD-CT and XRF-CT reconstructions in cortical bone. Ca XRF-CT 3D renders in (*a*) and (*b*) and XRF-CT slices in (*c*) and (*d*). (*e*) Examples of reconstructed diffractograms originating from the points marked with circled crosses in *f*. (*f*) Rietveld scale factor (*f*) and integrated background (*g*). Reproduced from Palle *et al*. [148] with permission from Elsevier.

One of the best XRD-CT resolutions recorded in bone to date is a sub-120 nm dataset obtained by Palle *et al*. [148] (figure 5) using a 12.7 keV 32.3 × 30.5 nm^2^ beam on an *ex vivo* sample of cortical bone taken from a human iliac crest at ID13, ESRF, almost an order of magnitude smaller than the previous record low in bone reported by Wittig *et al*. [149] around a year prior at DESY. Achieving high resolution in XRD-CT requires the use of beam sizes smaller than the preferred voxel size while maintaining sufficient flux to facilitate data collection, which is challenging in biomaterials like bone where diffraction signals are generally weaker. In this study, high resolution was achieved using multilayer Laue lenses (MLL) in a crossed geometry. While not the smallest possible beam size (i.e. <25 nm [150]), the selected 32.3 × 30.5 nm^2^ beam size is noted to offer a practical compromise between working distance, flux and spot size. Such studies are important early steps in optimizing XRD-CT for biomedical applications, here specifically the description of a technique achieving less than 120 nm resolution for combined XRD/XRF-CT measurements of bone. However, it should also be noted that the sample selected (i.e. healthy cortical bone) represents perhaps one of the easier forms of biomaterial to interrogate with its high bone mineral density, thicker lamellar structure being sampled from a higher load-bearing area, and lack of hydration all contributing to an optimized diffraction signal. It remains to be seen whether current capabilities can achieve such high resolution on samples of greater clinical interest such as low bone density samples (as seen in osteoporosis, rheumatoid arthritis, metabolic disorders and endocrine disorders).

#### XRD-TT

Tensor tomography is useful in probing the orientation of bone, which is already known to form in a highly directional manner in response to applied loads. Ossification is a complex process that is only partially understood from a mechanistic perspective, so information regarding bone structure can inform a better understanding of osteogenesis and osteopathic disease processes. Grünewald *et al*. [47] were the first to use combined SAXS/XRD-TT to demonstrate evidence of new structural features in human bone samples, reporting localized heterogeneities in the orientation of HAp, which might be interpreted as either an additional mineral fraction, a preferentially aligned extrafibrillar fraction, or transverse stacking of mineral particles. Similar work was performed by Murer *et al*., where XRD-TT was used to visualize the orientation of carbonated HAp crystallites and collagen fibres in porcine condyles. Across a reasonably large 3 × 3 × 3 mm^3^ FOV, HAp was found to orient their *c*-axis towards the ossification front despite lower resolution and signal-to-noise [151]. Most recently, Grünewald *et al*. [50] used SAXS/XRD-TT to investigate Haversian canals in lamellar bone, showing that cement lines have different material properties including crystallite size and orientation compared with the rest of the bone matrix.

#### (ii) Soft tissue

##### SAXS-CT

SAXS has good utility in biosciences as it provides nanoscale resolution ranging from around 1–100 nm (compared with 0.1–1 nm with WAXS), a length-scale appropriate for the interrogation of biorelevant samples like nanoparticles, proteins, colloids and liposomes, and is capable of providing structural information on non-crystalline samples in partially ordered systems. As a tomographic method, SAXS-CT provides a practical compromise between the large field-of-view offered by lower-resolution methods and the high-resolution diffraction information of local methods. Of all XDS-CT methods, SAXS-CT offers the greatest potential in imaging soft tissue, although the lack of nanostructure and propensity for radiation damage via radiolysis remains a fundamental challenge.

Experimental SAXS-CT has not been widely practiced in soft tissue. One of the earliest examples can be attributed to Jensen *et al*. in [152], who performed SAXS-CT on murine gliosarcoma tumour biopsies at cSAXS, SLS achieving a voxel volume of 30 × 30 × 40 µm^3^ [152]. The same authors also applied the same beamline technique to a murine brain fixed in formalin to successfully map myelin sheath concentration and the periodicity, in a study aimed to demonstrate the method's utility in investigating the pathophysiology of neurodegenerative disease [153].

In recent years, one of the only applications of SAXS-CT to soft tissue is a study by Conceição *et al*., who compared three fixated human breast tissue specimens on the D02A-SAXS2 at the National Synchrotron Light Laboratory (NSLL), Brazil [154]. In this case, the traditional challenges associated with X-ray soft tissue analysis are again surmounted via sample preparation interventions rather than SAXS-CT technique development. Freeze-drying is used to render the soft tissue more amenable to X-ray irradiation, removing around 99% of water while maintaining the material's structure, thus improving SAXS contrast and minimizing susceptibility to beam damage. A reverse analysis of the SAXS-CT data with *a posteriori* data of the lattice parameter, molecular shape and packing of collagen fibrils and triglycerides is used to show variation in their expression and degree of packing across normal tissue, benign lesion and malignant lesion samples. This study represents a compelling use of SAXS-CT as it compares clinically relevant healthy tissue with malignant tissue across a practical 2 × 2 mm FOV. The destructive sample preparation required improves SAXS signal but also means that *in vitro* experimentation is excluded, although granted the method used here is not any more destructive than current staining methods used in clinical histopathology.

##### SAXS-TT

**Figure 6.**
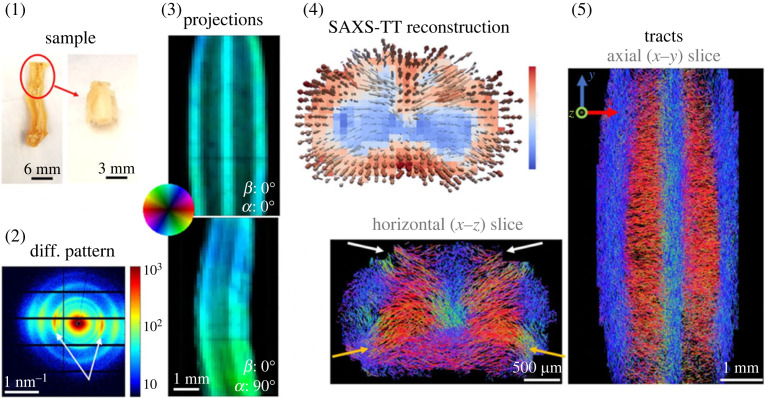
SAXS-TT exemplar in murine neuronal matter. Myelin peaks (2) from the cervical portion of a spinal cord (1) are scanned to produce two-dimensional SAXS projections (3), which are reconstructed to form tensor tomograms providing three-dimensional fibre orientations per voxel (4). Reproduced from Giorgiadis *et al*. [46].

More recently in 2021, Georgiadis *et al*. [46] used SAXS tensor tomography (SAXS-TT) to quantify myelin levels, axon orientations and nanostructural integrity in intact murine nervous tissue, demonstrating the technique's utility in interrogating demyelinating disease and axonal injury (figure 6). Looking forward, a computational study used simulated SAXS-CT to evaluate the potential for selectively differentiating amyloid targets in human and murine cerebral phantoms in a study attempting to image *β*-amyloid plaques *in vivo* without the use of tracers for the assessment of Alzheimer's disease [155].

### 3. Application of machine learning and deep learning

Within the field of ML and its subset deep-learning, convolutional neural networks (CNNs) have emerged as powerful tools for image analysis. CNNs, which sit within the broader class of artificial neural networks (ANNs), are structurally inspired by cerebral neural networks where different areas are connected via neurons. The mammalian neuron model is mimicked computationally with an architecture of data layers that processes input through a series of hidden layers to return an output value that has been transformed multiple times based on the weights of each layer node. Structurally, CNNs represent a more efficient version of ANNs that exploits the hierarchical nature of data to assemble input patterns in order of complexity as a method of regularizing to prevent overfitting [156]. As a result, CNNs become more scalable for complex image processing tasks.

CNNs form the basis of most modern image recognition and object detection systems, with applied examples including facial recognition [157,158], video analysis [159] and self-driving cars [160,161]. Within the medical imaging sector, their predictive capacity has been eagerly identified as a potential method to expedite diagnostic radiology workflows by automatically detecting or even grading pathologies for triage, although in practice this is unlikely to be adopted by the public healthcare sector in the near future based on its inconclusive diagnostic and prognostic value.

#### (a) Reconstruction

Better tomographic reconstruction strategies are an area of ongoing development [162]. The rising popularity of advanced tomography methods (including XDS-CT, spectroscopic CT methods and combined multimodal studies) coupled with faster acquisition instrumentation has led to a dramatic increase in the size of tomographic datasets. The use of CNNs as modern alternative to this ‘big data' problem is gaining traction in tomographic image reconstruction [163]. Compared with existing methods, deep learning represents a wholly new approach that relies on neither mathematical or physical models and instead uses the statistical power of large datasets. An inherent requirement then is that training datasets must be of sufficient size and scope. However, if trained adequately, deep-learning networks have the potential to supersede the performance of mathematical models, which in practice often fail due to deviations between the theoretical model and real-world data.

A wealth of deep learning networks for tomographic reconstruction have flourished in the last five years [163]. A present limitation for their direct application in XDS-CT data analysis is their limited scalability with large datasets, being originally designed for only absorption-contrast CT datasets. In recent years, members of the XDS-CT community have sought to address this limitation themselves by developing their own problem-specific CNNs. The capacity to flexibly train CNNs on different source data means these examples are also applicable to other forms of complex tomography data beyond diffraction.

An early example of a CNN capable of directly reconstructing images from projections is AUTOMAP, which uses supervised learning to map between the sensor and image domain to create an image [164]. A comparative limitation of AUTOMAP is its poor scalability with increasing pixels, which has led to the development of alternative methods based on CNNs and generative adversarial networks (GANs) [165] such as GANrec by Yang *et al*. [166], tomoGAN by Liu *et al*. [167] and a hierarchical synthesis CNN by Wu *et al*. [168]. Dong *et al*. devised a lightweight, CNN called the SingleDigit2Image (SD2I) network capable of reconstructing a tomographic image from a sinogram [169]. Its parametric reduction allows the network to scale more efficiently with increasing sinograms for XDS-CT. In tests on experimental XR-CT and XRD-CT data, the CNN yields qualitatively better reconstructions than existing direct (i.e. FBP) and indirect (i.e. SART, SIRT, CGLS) methods and was also shown to suppress angular undersampling artefacts in real and simulated data with limited projection information, demonstrating its utility in salvaging non-ideal datasets.

#### (b) Phase identification and quantitative analysis

The wealth of phase and structural information contained within large XDS-CT datasets can make manual identification an incredibly arduous task. In order to enable more efficient XDS-CT analysis of samples with multi-phase compositions, CNNs again emerge as a more computationally efficient method of analysing complex tomographic profiles.

Brumbaugh *et al*. used simulated data to train a one-dimensional CNN to identify target materials in XRD-CT datasets [170]. Relative improvements in classification accuracy were observed compared with standard correlation-based approaches, which were verified on both simulated and real data. Several other classification CNNs capable of phase identification have also been reported with better performance than manual regression but these struggle to perform multiple predictions for multiple structural parameters.

**Figure 7.**
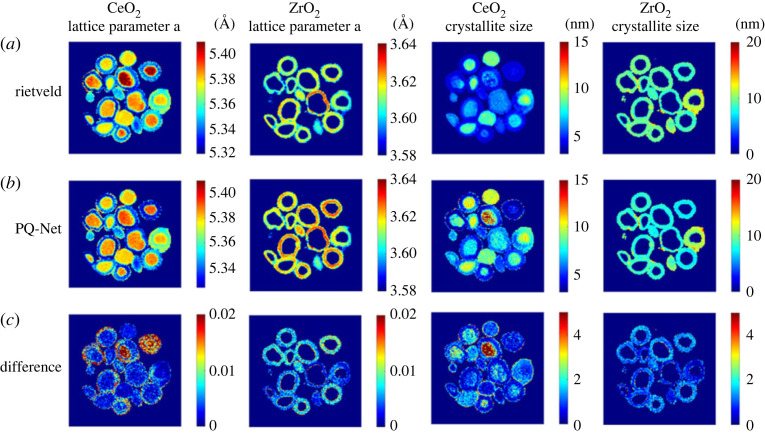
XRD-CT data analysis using the CNN PQ-Net in a model multiphase NiO-PdO-CeO_2_-ZrO_2_-Al_2_O_3_ catalytic system. Crystallite size and lattice parameter maps for CeO_2_ and ZrO_2_ obtained with the Rietveld method (*a*), results obtained with the PQ-Net (*b*), their absolute difference (*c*). Reproduced from Dong *et al*. [171].

Dong *et al*. developed a regression CNN termed parameter quantification network (PQ-Net) for the quantitative analysis of large multiphase XRD datasets [171]. The use of a regression rather than a classification CNN means the network is able to flexibly make predictions on a wider range of structural parameters implied in XRD profiles including lattice parameters, scale factors and crystallite sizes instead of making a binary decision on whether a phase is present or not. After training on libraries of simulated one-dimensional XRD profiles performance of PQ-Net was validated on a real dataset of a five-phase Ni-Pd/CeO_2_-ZrO_2_/Al_2_O_3_ catalyst consisting of over 20 000 diffraction patterns. Network predictions were comparable to that achieved via Rietveld analysis, shown in figure 7, with reliable uncertainty measures but in a fraction of the time. Its ability to produce results in a matter of seconds makes it a potential tool for real-time data visualization during *in situ* experimentation.

#### (c) Image and pattern enhancement

A handful of CNNs have also been developed to provide various enhancements that could be applied to XDS-CT datasets. A useful avenue of research that could be translated across to XDS-CT data are efforts in the medical imaging sector to improve image resolution or reduce artefacts with limited tomographic information [172,173]. These clinical motivation for these studies, which are often demonstrated on XR-CT, MRI or PET, is to reduce radiation dosage for patients and speed up acquisition times for higher patient throughput but remain potentially relevant for noise and limited tomographic information in XDS-CT datasets.

Hendriksen *et al*. presented a CNN for denoising multi-dimensional tomographic data in the absence of reliable reference data called Noise2Inverse [174,175]. Denoising for volumetric data is noted to be improved with the use of three-dimensional CNNs rather than two-dimensional CNNs although these are computationally heavy and time consuming. Denoising CNNs also typically use supervised learning, which can be prohibitive in cases where there is no ‘golden sample' or ideal ground truth dataset available for training. The Noise2Inverse method uses reconstructed training pairs where noisy images are mapped to each other. This is shown to work provided the noise in the reconstructed input image is statistically independent from noise in its target pair image and that all subsets of the measurement are used equally often in the target image. Validation on real datasets shows a qualitative increase in image quality and a quantitative reduction in analysis time.

## 4. Conclusion

To date, XDS-CT has found utility in a wide array of scientific fields including materials science, energy, catalysis, cultural heritage and biosciences. The diverse interest they have drawn over conventional imaging techniques can certainly be attributed to the wealth of volumetric chemical insight they can provide, which ranges from phase identification to quantification of structural parameters. Further, their capacity to be combined with *in situ* and *operando* techniques while still offering nanoscale resolution makes them ideally suited to fields with an interest in end-to-end characterization of evolving physico-chemical processes such as catalysis and energy sciences. Despite this, uptake is still arguably modest, which can be attributed to a few factors.

An experimental bottleneck that affects some situations more than others is beam damage. While generally classed as a non-destructive technique, examples in battery and biomaterial studies have shown where high-energy X-rays either damage or augment the evolving chemistry of the system. In the future this can be addressed with studies benchmarking optimal radiation dosing. However, in biomedical studies, a large amount of technique development would still be required as the high energy and flux of synchrotrons is fundamentally incompatible with *in vivo* experimentation. Application in biosciences will likely instead be advanced by the development of better laboratory-based XDS-CT systems. This would also address a related barrier to uptake, which is the globally limited access to synchrotron XDS-CT facilities.

However, the major bottleneck currently acknowledged by the XDS-CT community is the discrepancy between the capacity to acquire versus process data, with the sizable amounts of data being produced almost overwhelming traditional analysis methods. As demonstrated, deep learning methods are proving themselves to be the potential solution to this problem and will have a continued impact on the processing, analysis and collection of XDS-CT data in the future. It is likely that deep learning will enable the broader deployment of XDS-CT throughout research communities and help it to become a more mainstay chemical imaging technique.

## Data Availability

This article has no additional data.
